# Non-adherence and non-persistence to intravitreal anti-vascular endothelial growth factor (anti-VEGF) therapy: a systematic review and meta-analysis

**DOI:** 10.1186/s13643-023-02261-x

**Published:** 2023-06-02

**Authors:** Haris Shahzad, Sajid Mahmood, Sean McGee, Jessica Hubbard, Sayeed Haque, Vibhu Paudyal, Alastair K. Denniston, Lisa J. Hill, Zahraa Jalal

**Affiliations:** 1 University Hospitals Birmingham NHS Foundation Trust, Birmingham, UK; 2 Deputy Drugs Controller, Specialized Healthcare and Medical Education Department, Punjab Lahore, Pakistan; 3 School of Biomedical Sciences, Institute of Clinical Sciences, College of Medical and Dental Sciences, University of Birmingham, Birmingham, UK; 4 Institute of Applied Health Research, University of Birmingham, Birmingham, UK; 5 School of Pharmacy, Institute of Clinical Sciences, College of Medical and Dental Sciences, University of Birmingham, Birmingham, UK; 6 National Institute for Health Research (NIHR) Biomedical Research Centre at Moorfields Eye Hospital and University College London Institute of Ophthalmology, London, UK; 7 Academic Unit of Ophthalmology, Institute of Inflammation and Ageing, College of Medical and Dental Sciences, University of Birmingham, Birmingham, UK; 8 Department of Ophthalmology, University Hospitals Birmingham NHS Foundation Trust, Birmingham, UK; 9 Birmingham Health Partners Centre for Regulatory Science and Innovation, University of Birmingham, Birmingham, UK; 10 Health Data Research UK, London, UK

**Keywords:** Intravitreal, Anti-VEGF, COVID-19, Non-adherence, Non-persistence, Macular, Meta-analysis

## Abstract

**Background:**

Intravitreal anti-vascular endothelial growth factor (anti-VEGF) injections play a key role in treating a range of macular diseases. The effectiveness of these therapies is dependent on patients’ adherence (the extent to which a patient takes their medicines as per agreed recommendations from the healthcare provider) and persistence (continuation of the treatment for the prescribed duration) to their prescribed treatment regimens. The aim of this systematic review was to demonstrate the need for further investigation into the prevalence of, and factors contributing to, patient-led non-adherence and non-persistence, thus facilitating improved clinical outcomes.

**Methods:**

Systematic searches were conducted in Google Scholar, Web of Science, PubMed, MEDLINE, and the Cochrane Library. Studies in English conducted before February 2023 that reported the level of, and/or barriers to, non-adherence or non-persistence to intravitreal anti-VEGF ocular disease therapy were included. Duplicate papers, literature reviews, expert opinion articles, case studies, and case series were excluded following screening by two independent authors.

**Results:**

Data from a total of 409,215 patients across 52 studies were analysed. Treatment regimens included pro re nata, monthly and treat-and-extend protocols; study durations ranged from 4 months to 8 years. Of the 52 studies, 22 included a breakdown of reasons for patient non-adherence/non-persistence. Patient-led non-adherence varied between 17.5 and 35.0% depending on the definition used. Overall pooled prevalence of patient-led treatment non-persistence was 30.0% (*P* = 0.000). Reasons for non-adherence/non-persistence included dissatisfaction with treatment results (29.9%), financial burden (19%), older age/comorbidities (15.5%), difficulty booking appointments (8.5%), travel distance/social isolation (7.9%), lack of time (5.8%), satisfaction with the perceived improvement in their condition (4.4%), fear of injection (4.0%), loss of motivation (4.0%), apathy towards eyesight (2.5%), dissatisfaction with facilities 2.3%, and discomfort/pain (0.3%). Three studies found non-adherence rates between 51.6 and 68.8% during the COVID-19 pandemic, in part due to fear of exposure to COVID-19 and difficulties travelling during lockdown.

**Discussion:**

Results suggest high levels of patient-led non-adherence/non-persistence to anti-VEGF therapy, mostly due to dissatisfaction with treatment results, a combination of comorbidities, loss of motivation and the burden of travel. This study provides key information on prevalence and factors contributing to non-adherence/non-persistence in anti-VEGF treatment for macular diseases, aiding identification of at-risk individuals to improve real-world visual outcomes. Improvements in the literature can be achieved by establishing uniform definitions and standard timescales for what constitutes non-adherence/non-persistence.

**Systematic review registration:**

PROSPERO CRD42020216205.

**Supplementary Information:**

The online version contains supplementary material available at 10.1186/s13643-023-02261-x.

## Introduction

Therapies that inhibit vascular endothelial growth factor (VEGF), ‘anti-VEGFs’, play a key role in reducing angiogenesis and vascular permeability [1] with the aim to prevent sight loss in ocular diseases, including neovascular age-related macular degeneration (nAMD), diabetic macular oedema (DMO), macular oedema caused by retinal vein occlusion and myopic choroidal neovascularisation (myopic CNV). Currently, anti-VEGF treatments are exclusively administered to patients via intraocular injections for local retinal delivery of the drug. Randomised controlled trials (RCTs) and real-world studies have identified that anti-VEGF treatments, including Ranibizumab, Bevacizumab and Aflibercept [1–4] impart visual improvement to up to 40% of patients with nAMD, DMO and macular oedema and to about half of patients with myopic CNV [5].

Anti-VEGF treatment regimens can differ between patients with most patients requiring continual or even indefinite treatments. Patients typically receiving either proactive or *pro re nata* (PRN) approaches. Proactive treatment protocols involve regular anti-VEGF injections at fixed intervals, usually monthly. Prevention of further sight loss is still dependent on regular monitoring and patient adherence to their treatment regimens [6]. Previous studies have identified failure in adherence of patients to their anti-VEGF treatment regimens or follow up visits, with various reasons reported. These reasons include the frequency of required visits, difficulty in attending clinical and follow-up appointments, financial limitations, pain, disbelief in the benefit of the treatment, and refusal of continuance of treatment due to associated comorbid conditions [7–9].

The World Health Organization (WHO) defines adherence to long-term therapy as ‘the extent to which a person’s behaviour—taking medication, following a diet, and/or executing lifestyle changes—corresponds with agreed recommendations from a healthcare provider’ [10]. There are some differences to the definitions for ‘adherence’ and ‘persistence’ in the literature. Non-adherence, in patients receiving anti-VEGF therapies, would involve deviating from their prescribed therapeutic regimen. Persistence would typically define the duration of continuation with therapy [11], and non-persistence most often refers to patients choosing to stop their medication against the prescriber’s recommendation. Non-persistence definitions reported in patients on anti-VEGF treatments would typically include ‘discontinuation of therapy’ and ‘loss to follow up’, whereas non-adherence could refer to ‘missed appointments,’ ‘irregular attendance’ or ‘gaps in treatment’.

Medication non-adherence may occur at different points in a patient’s decision-making process. It may occur at the outset of their therapy or at some point during their therapy. Previous studies have reported a variation in the rates of therapy discontinuation (non-persistence) of anti-VEGF treatment in diseases such as nAMD to be approximately 42% [9] and 50% [12], with factors such as patients’ level of awareness of their disease and treatment affecting compliance to therapy. Similarly, the adherence to treatment regimen with anti VEGF therapy improves the clinical outcomes in patients with nAMD, DMO and CNV [5, 13–15].

Recently, the coronavirus (COVID-19) pandemic has impacted patient-led adherence to intravitreal injections [16]. The governments around the world imposed strict measures to prevent the spread of the disease. This included stay-at-home advisories and a reduction in non-urgent care [17, 18]. The consensus among retinal disease experts was that for neovascular AMD, retinal vein occlusion and diabetic retinopathy patients, anti-VEGF injection regimens should continue during lockdowns or curtailed non-urgent ophthalmic services [19]. Regardless, from a patient perspective, fear of infection, difficulty travelling, and COVID-19 infection within a household were likely to have had an impact on attendance to appointments [20, 21]. It is therefore crucial to quantify levels of non-adherence during the pandemic to inform future practices to minimise disruption to essential ophthalmic care.

Recent systematic reviews have investigated patient non-adherence and non-persistence to anti-VEGF treatment regimens in nAMD and DMO specifically [12, 22, 23]; all identify a need for further investigation in this understudied area. In particular, there is a need to investigate reasons for non-adherence and non-persistence, rates of attendance for follow-ups and to determine strategies to tackle these challenges of under-treatment and reduce the burden of ‘sight-threatening’ chronic eye diseases for patients and healthcare providers. The aim of this systematic review and meta-analysis was to investigate the prevalence of patient-led non-adherence/non-persistence to intravitreal anti-VEGF therapy, and the barriers/reasons associated with non-adherence/non-persistence in different disease states.

## Materials and methods

### Ethics

No ethical approval was required for this systematic review. The PRISMA (Preferred Reporting Items for Systematic Review and Meta-analysis) guidelines were strictly followed in our reporting.

### Review registration

The review was prospectively registered on the PROSPERO database of systematic reviews (CRD42020216205).

### Literature searches

Literature searches were conducted between December 2020 and February 2023. Covidence® (Cochrane, Melbourne, Australia) was utilised for management and screening of systematic reviews.

### Inclusion and exclusion criteria

Clinical studies eligible for inclusion were those that detailed either the level of non-adherence or non-persistence to medication and therapy follow-up, among adult patients (18 years old and above) with any ocular disease requiring anti-VEGF therapy. There were no exclusion criteria regarding the definitions used of either non-adherence or non-persistence. There were no eligibility restrictions based on the type of anti-VEGF agent used, treatment regimen or type of ocular disease. There were no restrictions on the setting for the anti-VEGF treatment being administered. Studies that were reported in languages other than English were excluded. Reviews (both systematic and narrative), expert opinion articles, case studies and series were excluded. Studies that did not report either non-adherence or non-persistence outcomes were not included. Studies in which patients received intravitreal injections that were not anti-VEGF agents were also excluded.

#### Search strategy and study selection criteria

Electronic literature searches were conducted in Web of Science, PubMed, MEDLINE, PsychINFO, Cochrane Library (The Cochrane Database for Systematic Reviews), CINAHL Plus (A Cumulative Index to Nursing and Allied Health Professionals) and Google Scholar. The search terms used in our data search were (adherence or non-adherence or persistence or non-persistence or dropout or continuation or discontinuation or lost to follow up or loss to follow up or LTFU or cessation or persistence or non-persistence or undertreatment or compliance or non-compliance or missed appointments or irregular attendance or treatment gaps) and (age-related macular degeneration OR AMD or wet AMD or neovascular AMD or nAMD or diabetic macular oedema or DMO or diabetic macular edema or DME OR diabetic retinopathy OR proliferative diabetic retinopathy OR PDR OR retinal vein occlusion OR RVO OR BRVO OR CRVO OR choroidal neovascularization OR CNV) AND (Anti-VEGF OR anti vascular endothelial growth factor OR ranibizumab OR Lucentis OR Aflibercept OR Eylea OR Zaltrap OR Avastin OR Bevacizumab OR Brolucizumab OR Pegaptanib OR Antiangiogenic).

#### Study selection and data extraction

Search results were imported into Covidence®. Titles, abstract and full text of potential studies were downloaded and assessed against our inclusion exclusion criteria by two authors independently. Any disagreement was resolved by a third reviewer. A data extraction sheet was created using MS Excel®. The parameters extracted from each of the selected studies include the study title, name of investigator, year of study, country of study, study design, duration of study, sample size, gender distribution, participants in the study, mean age of participants, intervention and regimen used in the study, definition of non-adherence/non-persistence used in the study, overall level of non-adherence/non-persistence among the participants, patient associated non-adherence/non-persistence and reported reasons for non-adherence/non-persistence to the prescribed anti-VEGF therapy.

#### Main outcome(s)

These are the rate/prevalence of patient-led non-adherence to anti-VEGF therapy and the rate/prevalence of patient-led non-persistence to ant-VEGF therapy.

Factors/reasons/barriers associated with non-adherence were analysed using the World Health Organization’s multidimensional adherence model (MAM). We categorised factors associated with non-adherence by patient-related, healthcare system-related, condition-related, and treatment-related factors.

#### Data quality assessment

The National Institutes of Health (NIH) quality assessment tool was used to assess the quality of included studies. This scale assesses the quality of both cross sectional and observational cohort studies and consists of 14 questions. For each question three options, ‘’Yes’’, ‘’No’’ and ‘’Not Applicable’’ are available for the reviewer to choose as appropriate. Depending upon these questions the studies were categorised as ‘Good’, ‘Fair’ or ‘Poor’ Quality by two reviewers. In the event of review discrepancies between these reviewers, a third reviewer was consulted to resolve the difference.

#### Statistical analysis

Statistical analysis was performed by using STATA© Version 14. A meta-analysis on patient-led rates of non-persistence to treatment was undertaken. Non-persistence was used instead of non-adherence given the heterogeneity of non-adherence definitions. The purpose of assessing patient-led rates was to compare and quantify the rates of non-persistence to treatment where the primary decision-maker to cease treatment or not attend follow-up was the patient, as opposed to the healthcare provider or some external factor. Excluded reasons for non-adherence or non-persistence included patient death, futility, remission, transfer of care elsewhere, and administrative error, among others. Although there was some variation between definitions of non-persistence between studies, it was feasible to pool the patient-led non-persistence outcomes given the similarity between definitions such as discontinuation. A random effects model was used for the estimation of non-persistence rate among the patients to rule out the presence of high heterogeneity among the included studies. *I*^2^ (% residual variation due to heterogeneity) test with 95% confidence interval was used to estimate statistical heterogeneity. *I*^2^ value of ≤ 50 was used to indicate statistical homogeneity. Furthermore, subgroup analysis was performed to find out the difference in persistence rate among the patients who discontinued within one year of treatment and those who discontinued after one year of treatment.

## Results

**Fig. 1:**
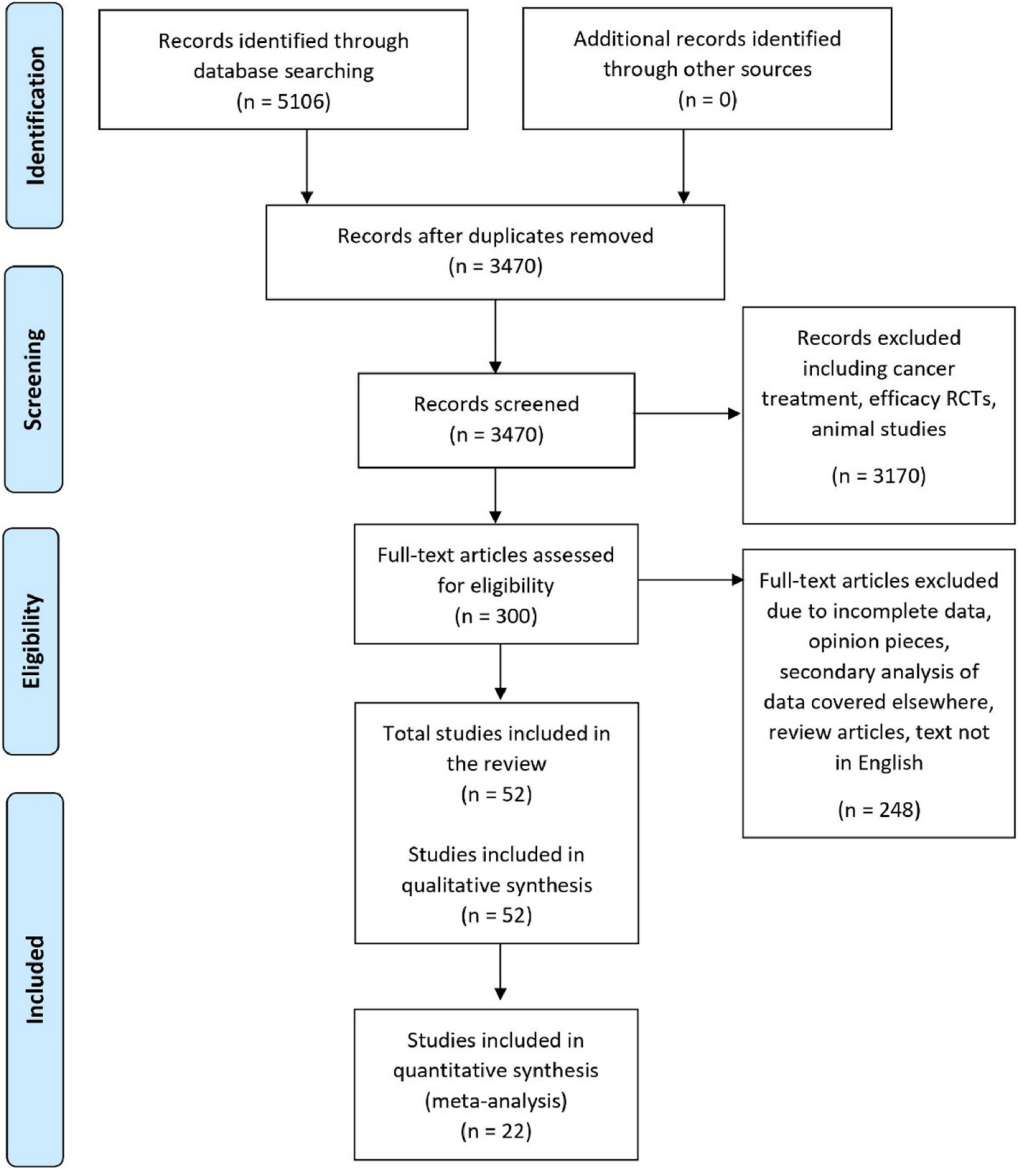
Preferred Reporting Items for Systematic Reviews and Meta-Analyses (PRISMA) flow chart. Illustrates the number of records identified, screened, and excluded at each stage

A total of 5063 studies were retrieved from the databases after searching for the key terms. Once duplicates were removed, 3470 studies remained. After screening titles and abstracts for relevance and exclusions, 300 potential articles remained for full-text review. Following the full assessment, a total of 52 studies remained eligible and were included in the analysis. Research measuring non-adherence or non-persistence to anti-VEGF therapy in adult patients for nAMD, DMO, CNV or macular oedema following RVO was included. A Preferred Reporting Items for Systematic Reviews and Meta-Analyses (PRISMA) flow chart illustrates the number of records identified, screened and excluded at each stage (Fig. 1).

### Study characteristics

Of the 52 studies deemed eligible for inclusion and full analysis, 22 included a breakdown of the associated reasons underlying patient non-adherence and non-persistence [8, 9, 13, 24–42]. Eleven studies reported barriers to treatment and patient-related factors associated with under-treatment without a breakdown of reasons [15, 43–52]. Nineteen studies gave rates of non-adherence or non-persistence without detailing either associated factors, reasons, or barriers to treatment [53–71].

### Study design

The majority of studies included here were retrospective, with 6/52 studies having an element of prospective data collection [24, 28, 37, 45, 60, 63]. Nunes et al. [32] was a retrospective case series, and studies by Ramakrishnan et al. [38, 62] were retrospective analyses of an RCT. The remaining studies were retrospective observational research investigating adherence outcomes from patient treatment records. Twelve studies inquired about the reasons for treatment irregularity or discontinuation from intravitreal regimens through patient interviews in person or over the phone [8, 13, 25, 29, 31–33, 35, 42, 45, 48, 54]. In addition to data collection on non-adherence and non-persistence, a number of studies also used patient factors such as age, distance from treatment centres, ethnicity, their first language and visual acuity to determine whether any factors correlated with adherence [15, 43–46, 48, 50–52].

### Quality assessment

The majority of studies 41/52 (78.85%) included in our review were rated as of ‘Good’ quality on NIH quality assessment tool. The remaining 9/52 (17.3%) [8, 9, 26, 31, 44, 56, 62, 63, 68] studies were rated as of ‘Fair’ quality and one study was rated ‘Poor’ [25].

### Locations

Included studies were set in Germany (6 studies), USA (8 studies), Australia (4 studies), France (4 studies), Austria (4 studies), UK (3 studies), Denmark (2 studies), Sweden (2 studies) and Egypt (2 studies), as well as Turkey (2 studies), Israel (2 studies), Singapore (1 study), Japan (1 study), Brazil (1 study), Italy (1 study), Taiwan (1 study), India (1 study), Canada (1 study), Finland (1 study) and Jordan (1 study), whereas 4 studies were conducted in more than one country.

### Patients/disease groups

Forty-three of the 52 studies assessed non-adherence and/or non-persistence among nAMD patients receiving intravitreal anti-VEGF treatments. Fifteen studies were conducted on patients with proliferative diabetic retinopathy/diabetic macular oedema [15, 24, 29, 35, 38, 41–45, 54, 55, 59, 64, 66]. Two studies included patients receiving anti-VEGF treatment for macular oedema following retinal vein occlusion [15, 29]. Overall, 409,215 patients were included in our study, with the largest study involving a retrospective chart review of over 194,000 Medicare beneficiaries in the US [57]. The gender distribution was roughly balanced, with proportion of males ranging from 33% [65] to 65% [66].

### Interventions

Thirty-five studies assessed patients who had been treated with intravitreal Ranibizumab injections [8, 9, 13, 15, 25–31, 33–39, 41, 43, 47–51, 53, 55–58, 60–62, 70, 71]. Twenty-one studies assessed patients taking Aflibercept injections [15, 26, 34–36, 38, 39, 41, 43, 45, 49–52, 63–66, 68, 70, 71]. Seventeen studies included Bevacizumab as the intravitreal drug [29, 32, 35, 38, 39, 41, 45, 46, 49–51, 57, 61, 62, 69–71], and three studies looked at patients treated with Pegaptanib sodium, all anti-VEGF agents [45, 48, 57]. Eight studies did not specify the drug used for intravitreal treatment [24, 40, 42, 44, 54, 59, 67, 69]. The dosing schedules varied depending on the anti-VEGF agent used and the local protocols. Regimens included three loading doses in the first month plus pro re nata (PRN) maintenance treatment every 4–6 weeks, PRN, 2 monthly, single injections followed by PRN, and treat-and-extend protocols.

### Study duration

Thirty-two studies had a duration of up to 3 years [8, 13, 15, 25–27, 31–34, 36–38, 41–43, 45, 48, 53–60, 62, 64, 67–69, 71]. Twenty studies had a duration of over 3 years [9, 15, 24, 28–30, 35, 39, 40, 46, 47, 49–52, 61, 63, 65, 66, 70]. The studies with the longest duration over which data was collected investigated discontinuation over an 8-year period [51]. The oldest studies collected patient data from 2006, and the latest data sets were during 2022.

### Assessment of outcomes, definitions

Across all studies analysed in this project, there was a diverse range of measures of non-persistence such as ‘discontinuation’, ‘incompleteness of follow-up’, ‘loss to follow-up’, ‘cessation’ and ‘drop-out’. Non-adherence was referred to as ‘irregular attendance’, ‘unintended treatment gaps’, ‘missed appointments’, ‘skipped doses’ and ‘delayed injections.’ Additionally, there was no widely accepted threshold for what determined non-persistence or non-adherence, nor a standard timescale in which to label non-adherence or non-persistence. This variety of definitions served as a challenge when comparing results between studies as a result of heterogeneity in outcomes, highlighting the need for establishing standards of what constitutes non-adherence and non-persistence in the context of anti-VEGF injection regimens. Two studies failed to clearly define what they considered to constitute non-adherence or non-persistence, referring to whether ‘local guidelines’ were followed or not [56] or ‘any deviation from regular treatment’ [63]. Several studies measured non-persistence after approximately 1 year.

Overall, non-persistence was measured as discontinuation at various time points such as 12 months, 2 years, the study period, incomplete 1 year follow-up, visit-free intervals of more than 6 months, visit-free interval of 12 months from the last injection, absence of patient follow-up after 3 months from the last appointment, missing any follow-up visit for an interval exceeding 6 months, having a termination visit, and not re-injecting despite best corrected visual acuity (BCVA) loss of more than 5 letters.

Definitions of non-adherence had even greater variation in terminology used including extent of irregular attendance by exceeding a 4-week follow-up by more than 2 weeks, more than 60 days between visits, unintended treatment gaps of more than 8 weeks, at least one missed appointment, skipped injections, delayed or dropped appointments in the first year, any deviation from the European guideline of 3 monthly doses followed by once every 2 months for 12 months, treatment gaps over 6 months, delayed follow-ups longer than 4 weeks, or missing any of the 3 monthly loading doses.

For some studies, reasons for non-adherence or non-persistence were ascertained through patient notes or interviews. As a result, external factors such as patient deaths or physician-led decisions to stop treatment due to futility or treatment success for instance could be differentiated from the patient-led factors.

### Prevalence of non-adherence

Given the variety of definitions used for non-adherence, a meta-analysis on adherence was not possible. The levels of overall non-adherence to intravitreal injection visits varied between 15.0% [54] and 95.6% [56], with patient-led non-adherence varying between 17.5 and35.0% depending on the definition used [15].

For instance, Massamba et al. [60] evaluated the impact of summer vacation on visual acuity of nAMD patients treated with intravitreal Ranibizumab. They defined non-adherence on the basis of opting to skip an injection during their holidays and found that 33 (53.2%) patients had skipped one or more injections during the break. Ramakrishnan et al. [62] assessed the association of visit adherence to visual acuity in nAMD patients. Non-adherence was measured as the number of days between visits. They found that 208 (17.7%) of patients had at least one period where no visit had occurred for more than 60 days. Abu-Yaghi et al. [54] defined non-compliance as missing either the three loading doses or any prescribed injections in the 12-month study period. Eighteen (15%) patients were classed as non-compliant based on these parameters. Once again, this highlights the limitations of a lack of consensus regarding what comprises non-adherence or non-compliance. In a study by Cohen et al. [56] for example, non-adherence was not clearly defined, with a range of measures mentioned such as whether patients were monitored every 30 days ± 7 days, whether guidelines were followed or not, and whether patients had regular attendance at least every 51 days. Of the 551 patients included, none were monitored every 30 days (± 7 days) for the duration of the study period, and 527 (95.6%) of patients had not been seen every 51 days. Such strict measures of adherence would therefore overestimate the prevalence of non-adherence.

### Prevalence of non-persistence

Patient-led rates of non-persistence, in which the primary decision-maker to cease treatment or not attend follow-up was the patient, were between 2.9 and 43%. Again, this varied according to the definition used. Angermann et al. [43] investigated treatment compliance among diabetic retinopathy and nAMD patients treated with Ranibizumab or Aflibercept from 2015 to 2018. Lost to follow-up was defined as a visit-free interval of more than 6 or 12 months, without the appointment being rescheduled. With this definition, the rate of discontinuation for the subset of 841 nAMD patients was 2.9%. This could be partly explained by the setting—conducted under universal healthcare coverage in Austria. Although there was no breakdown of reasons, age over 70 and a need for assisted transport were associated with discontinuation. Ng et al. [61] on the other hand, found a discontinuation rate at 12 months of 39.5% in their study of nAMD in Singapore in 2011, where there is a greater emphasis on individual payment for treatments in combination with government subsidies. Periods also varied between study definitions, with a paper by Vaze et al. [9] in 2014 defining non-persistence as permanent discontinuation within a 6-year period. Perhaps unsurprisingly, total discontinuation was found to be as high as 105 (42.3%), although the patient-led discontinuation was 26 (10.5%). Given the overlapping diseases investigated in different papers, such as Ehlken et al. [15] looking into nAMD, RVO, and DMO and combining the results, or other studies investigating one or two diseases and separating the results [35, 55], it was not possible to assess the differences in non-adherence or non-persistence levels between the disease states.

### Reasons for non-adherence/non-persistence

**Fig. 2:**
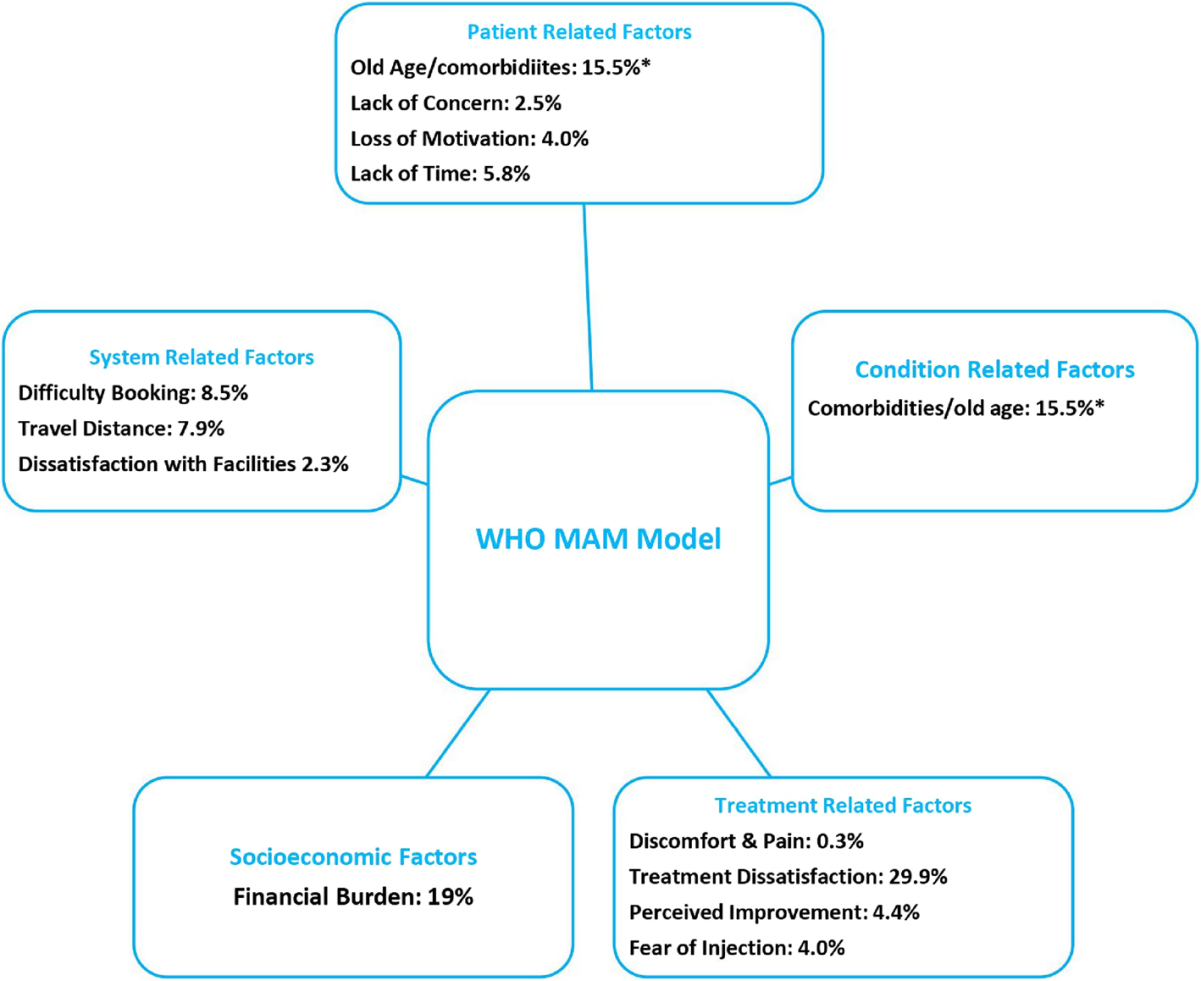
Reasons for non-adherence and non-persistence provided by patients identified in this review. Findings are sorted by patient-led, treatment-led, and other factors as provided by WHO MAM Model. In total, 937/409,215 provided patient-led reasons, out of which treatment dissatisfaction (29.9%), financial burden (19.0%) and old age/co-comorbidities (15.5%) were the most commonly occurring reasons provided. *Old age and co-morbidities were given as a single percentage of 15.5% due to the fact that these were combined in several of the papers. Aside from patient-associated factors given, several studies included physician-associated factors and external factors such as patient death and transfer of care elsewhere

In total from all studies, 937/409,215 provided patient-led reasons why they discontinued or were non-adherent. Reasons included dissatisfaction with treatment results 29.9%, old age/comorbidities 15.5%, difficulty booking appointments 8.5%, travel/distance/social isolation 7.9%, lack of time/job/family commitments 5.8%, perceived improvement 4.4%, fear of injection 4.0%, loss of motivation/burden of frequent visits 4.0%, lack of concern regarding eyesight 2.5%, dissatisfaction with facilities 2.3%, discomfort/pain 0.3%, and other reasons such as personality clash with physicians or unspecified personal reasons 1.9% (Fig. 2).

Additionally, a study by Sobolewska et al. [39] explored barriers underlying patient non-adherence in patient groups of differing follow-up time, using the Adherence Barriers Questionnaire Intravitreal Therapy (ABQ-IVT) [72]. This study found the following barriers to anti-VEGF therapy: time commitment (68.5%), challenge accompanying person to doctors’ appointments (57.4%), burden for family members (50.0%), travel/opportunity costs (46.3%), financial burden of treatment (42.6%), comorbidities (24.1%), depression (20.4%), dissatisfaction with treatment results (18.5%), belief in need for therapy (16.7%), shared decision making in treatment course (16.7%), uncomfortable feeling in doctors’ office (14.8%), side effects (13.0%), knowledge about therapy (13%), trust in physician (11.1%), lack of support (11.1%), too old for therapy (11.1%) and private/professional obligations (5.6%). While this study explores a range of barriers, it could not be included in the calculations listed above as the format of the study would have led to individuals’ responses to separate barriers being included twice in our sub-categories.

One of the most frequently reported factors was distance from the hospital or burden of repeated travel. In a 2017 study by Subhi et al. [34] in Denmark, patients were offered free of charge transportation which may have affected discontinuation. A key system-related factor was financial. For example, in a 2017 study by Polat et al. [13], patients in Turkey must pay a proportion of the price of Ranibizumab despite health insurance. Of the 314 patients included, financial difficulty was given as a reason for non-adherence in 8.3%. A study completed in Austria [3], however, was conducted in the setting of universal health coverage and had a low overall rate of non-adherence at 2.9%. In a study by Obeid et al. [47], certain factors were found to be correlated with lost to follow-up: older age, greater distance to clinic and unilateral eye disease. This suggests that age and transportation may be associated with the burden of treatment and that when patients have one unaffected eye, they may be more inclined to stop treatment early.

### Meta-analysis

**Fig. 3:**
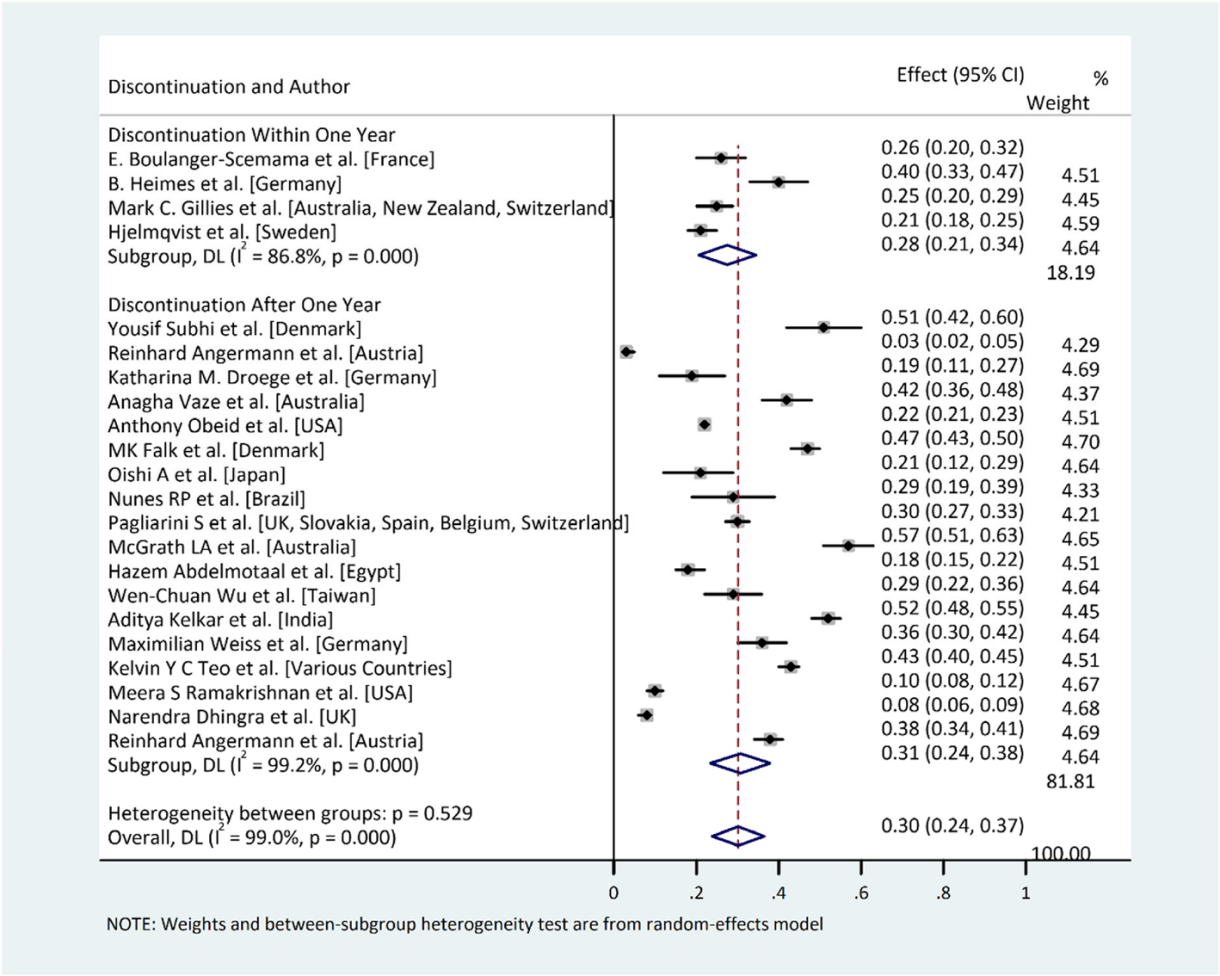
Meta-analysis results. Prevalence of patient-led treatment non-persistence among the patients on anti-VEGF therapy and subgroup analysis within 1 year of non-persistence to the prescribed anti-VEGF therapy according to duration. The overall prevalence of non-persistence at this time point is 0.3 (i.e., 30%)

The results of our meta-analysis on patient-led non-persistence revealed that the overall pooled prevalence of non-persistence among the patients on anti-VEGF therapy was 30% (overall non-persistence was 24–37%; *P* = 0.000). Similarly, the results of subgroup analysis revealed that there is no significant difference (*P* = 0.529) in risk of patient-led non-persistence to the prescribed anti-VEGF therapy during the first year of treatment as 28% (overall non-persistence 21–34%) patients left the treatment during first year as compared to the patients who left the treatment after 1 year 31% (24–38%). High heterogeneity (*I*^2^ = 99%, *P* = 0.001) was observed among the included studies (Fig. 3). This could be due to the greater variation in population characteristics, social and cultural variations among the participants as the studies included in this study were from different parts of the world.

### Prevalence of non-adherence during the COVID-19 pandemic

Three studies on patient adherence to anti-VEGF treatments during the COVID-19 pandemic met the inclusion criteria after being identified in the literature search. This included a 2022 paper based in the USA by Douglas et al. [67], a 2022 paper based in Israel by Arnon et al. [69] and a 2021 article from Turkey by Sevik et al. [68]. All three studies were retrospective in nature. Arnon et al. and Sevik et al. investigated 77 and 104 nAMD patients, respectively, whereas Douglas et al. included 1001 patients with nAMD (*n* = 579), diabetic retinopathy (*n* = 208), and retinal vein occlusion (*n* = 214). The 2022 USA study included patients from December 2019 to December 2020. The Israel study and Turkey study included data from March 2020 to April/June 2020. The mean age of patients from all three studies was between 75 and 80 years old. The 2021 Turkey study [68] involved a treat-and-extend protocol rather than a 3 monthly loading dose followed by monthly injections. In all three studies, patients were defined as non-adherent on the basis of cancelled or missed appointments. In addition, patients who were late to visits were also classed as non-adherent in the 2022 Israel study [69].

Overall, non-adherence rates were high: 51.1%, 68.8% and 57.7% in the USA, Israel and Turkey studies, respectively. In addition, the USA study found that the delay in appointment for those patients who missed their intended follow-up was 59 days. Furthermore, this group of patients had a statistically significant reduction in best-corrected visual acuity compared to the adherent group. These results corroborated with both the Israel and Turkey study regarding visual outcomes. Moreover, the 2021 Turkey study found a significant worsening of anatomical outcomes in nAMD as measured by optical coherence tomography at baseline and follow-up. Although none of the studies included a breakdown of the specific reasons for non-adherence, they alluded to the established patient-factors associated with poorer compliance, such as old age and comorbidities.

## Discussion

The findings of this systematic review and meta-analysis demonstrate relatively high levels of patient-led non-adherence and non-persistence to intravitreal anti-VEGF therapy for a variety of macular diseases and identifies key factors contributing to these events.

The level of non-adherence to intravitreal injection visits was high yet varied depending on the definition applied. Overall non-adherence was measured as high as 95.6% based on a definition utilised by Cohen et al. [56]. In contrast, 15.0% of patients were defined as non-compliant by Abu-Yaghi et al. [54], with non-compliance defined as missing either the three loading doses or any prescribed injections in the 12-month study period. Given the heterogeneity in measures of non-adherence, an accompanying meta-analysis was not conducted.

It is interesting to note that poorer baseline visual acuity and patient dissatisfaction with treatment outcome were both associated with non-adherence, which, in turn, leads to poorer visual acuity. Given the chronic nature of retinal diseases and the frequent need for several courses of anti-VEGF treatment to achieve noticeable visual improvements, dissatisfaction with initial outcomes may be a result of unrealistic expectations. Further dissatisfaction with treatment outcome and patient-experience drives a self-perpetuating cycle ultimately leading to vision loss.

The results of our meta-analysis into patient-led non-persistence revealed that the overall pooled prevalence of treatment non-persistence among the patients on anti-VEGF therapy was 28.0% (21.0, 34.0%) at 12 months and 31.0% (24.0, 38.0%) in studies lasting longer than 12 months. Given that non-persistence rates were similar in studies lasting 12 months and those lasting longer than 12 months, this suggests that once a patient completes a year-long course of intravitreal injections, they are less likely to discontinue in the near future. Thus, the vast majority of non-persistence occurs within a year of starting treatment, indicating that the decision to discontinue, based on patient-led factors, is often made early on in the course of treatment.

The reasons for this are likely to be multifactorial, with factors such as perceived improvement, establishing routine, and overcoming initial barriers explaining why those who remain on treatment for one year seem more invested in continuing treatment.

Reasons provided and factors associated with non-adherence and non-persistence were multifactorial, with socioeconomic, patient experience, and healthcare system factors identified. The most prevalent reasons for discontinuation or attendance irregularity were dissatisfaction with treatment results (29.9%), financial burden (19.0%), old age/comorbidities (15.5%), difficulty booking appointments (8.5%) and travel distance (7.9%). Additionally, certain factors were found to be correlated with loss to follow-up: older age, greater distance to treatment centre, poorer baseline visual acuity and unilateral eye disease.

Noteworthy reasons commonly provided for non-adherence and non-persistence include the associated financial burden experienced by the patient, elevated average age and comorbidities. Comparing studies conducted in countries of contradicting financial obligations, for example Angermann et al. [43] in Austria (has a universal healthcare cover system) and Ng et al. [61] conducted in Singapore (individuals are expected to contribute to treatment expenses), enables investigation into the impact of socialised healthcare on treatment continuation. The rate of discontinuation for nAMD patients in the Austrian study was found to be as low as 2.9%, comparatively, Ng et al. [61] identified a patient-led discontinuation rate at 12 months of approximately 40% in Singapore. This difference indicates the significant influence of financial demands in treatment persistence.

Given that the majority of studies included in this review list old age and comorbidities among the top factors associated with non-adherence or non-persistence, the impact of these appear significant and warrants further investigation. Travel distance and social isolation were additional contributing factors in several studies, potentially compounded by the elderly populations frequently affected by retinal disease. No studies elaborated on what the comorbidities entailed or how they contributed specifically to patient attendance at appointments, therefore emphasising the need for researchers to introduce sub-categories that encourage specificity when referring to comorbidities.

The studies in our review reported non-adherence rates which ranged from 15.0% [54] and 95.6% [56], and this range of values is consistent with a review by Okada et al. [12] which demonstrates a similar range of values (32–95%) for AMD patients. The overall pooled prevalence of non-persistence among patients on intravitreal anti-VEGF injections in our review was 28.0% (21.0–34.0%) at 12 months. These values offer a similar range to Okada et al. [12] (3–57%).

The wide range in non-adherence levels reported between studies is associated with considerable variation in how non-adherence was defined. There was, however, reasonable congruence when similar definitions for non-adherence were used, such as the studies by Abu-Yaghi et al. [54] and Habib et al. [45] for which non-adherence was reported as 15% and 20.7%, respectively.

Additionally, our review found that a variety of socioeconomic, patient experience and healthcare system factors contributed towards non-adherence and non-persistence to therapy. The contributing factors found by our study, mentioned previously, are highly consistent with the findings of Okada et al. [12]. This study specifically demonstrated that factors such as baseline visual acuity, lack of transport/distance to treatment centre, financial burden including indirect travel costs as well as many others contribute to non-adherence and non-persistence.

Furthermore, our review identified three studies on patient adherence to intravitreal injections during the COVID-19 pandemic. Overall non-adherence rates were identified as between 51.1 and 68.8%, along with poorer adherence linked to a statistically significant worsening in functional and anatomical outcomes [67–69]. Elsewhere in the literature, numerous studies report factors associated with reduced adherence to anti-VEGF injections during the pandemic. A study by Viola et al. based in Milan, Italy, [21] found poor compliance associated with periods of lockdown, better vision in the untreated eye and older age. A study based in Germany [20] suggested fear of exposure to COVID-19, difficulties travelling during lockdown, older age and COVID-19 infection in the family as added challenges of attending intravitreal injection appointments during the pandemic. Retinal disease experts emphasise the importance of balancing eyecare and patient safety when continuing anti-VEGF injections during COVID-19 outbreaks. Triaging retinal disease patients to identify and prioritise those at greatest risk of sight loss has been suggested as a consideration focus to minimise the risk of patient and staff exposure to COVID-19 [19].

This review has several strengths. While other reviews have investigated both the prevalence and the factors contributing to non-adherence and non-persistence, they have not included a meta-analysis. This is the first meta-analysis of non-persistence in anti-VEGF therapy and allowed for sub-group analysis. The scale and quality of the data also provides confidence in the findings with 52 studies identified most of which were rated as good quality. These 52 studies were based in 24 countries and included 409,215 patients. The included studies assessed a range of macular diseases, multiple intravitreal anti-VEGF therapies and a variety of treatment regimens. However, this study is not without limitations. The majority of studies included in this review were based in countries with predominantly Caucasian populations, limiting the applicability of our findings. The complexity of some significant factors may also be hidden by broad categories such as ‘Comorbidity’; it would be impossible to plan policy interventions to address this barrier without having more detail on this. For example, the presence of a concurrent eye disease that may alter the patients view on treatment effectiveness leading to reduced patient compliance to therapy. In comparison, the impact that a mobility-limiting condition will have on treatment adherence would be different and may be more easily addressed. A significant issue encountered in this review is the heterogeneity of definitions for non-adherence and non-persistence used across included studies. This heterogeneity prevented meta-analysis of non-adherence and makes the identification of contributing factors, at-risk individuals and the development of targeted treatment plans more difficult.

Many implications for practice have arisen from the findings of this review. One of the major factors found to be associated with non-adherence and non-persistence was patient dissatisfaction with treatment results. Given that multiple courses of anti-VEGF treatment are frequently required to achieve noticeable improvements in vision, patient dissatisfaction may be the result of unrealistic expectations. The introduction of pre-treatment education, aiming to set realistic expectations for treatment outcome, might aid therapy continuation.

Another highly cited reason for non-adherence and non-persistence to therapy was travel distance and social isolation, a significant issue among the elderly populations affected by retinal disease. Travel bursaries could ameliorate this barrier to therapy, as demonstrated by Okada et al. [12]. Financial burden, including non-direct costs, was one of the most commonly listed reasons for patient-led discontinuation.

A variety of healthcare system-related factors were implicated in this review. These factors include difficulty booking and dissatisfaction with facilities. While these issues are only a fraction of the reasons patients gave for non-adherence and non-persistence, they indicate the strain that ophthalmic healthcare systems are currently under. With an ageing population and high myopia expected to affect 9.8% of the global population by 2050 [73], ophthalmic disease and the strain on healthcare systems is only going to rise, indicating that the growing demand on administrative factors and difficulty in acquiring appointments will only continue and worsen. This underlines the need for sufficient resource allocation to prevent unnecessary treatment delays under a growing patient pool, to prevent vision loss.

This review has demonstrated that old age and comorbidities have a large influence on the adherence and persistence to therapy. This highlights the need for more flexible treatment regimens such as the treat-and -extend protocol that reduces the burden of treatment, in line with patients’ ability and willingness to attend regular anti-VEGF appointments. Further understanding of the comorbidities that present a high risk of non-adherence and non-persistence to treatment is needed to accurately identify at-risk individuals; this emphasises the need for future researchers to introduce sub-categories to allow for more specific data collection regarding the relationship between specific comorbidities and non-adherence and non-persistence to therapy.

## Conclusion

Our review is the first systematic review with meta-analysis to examine the prevalence and factors associated with patient-led non-persistence to intravitreal anti-VEGF therapy in the treatment of retinal disease. Our findings show high levels of both non-adherence and non-persistence to treatment therapy, due to a variety of socioeconomic, patient experience and healthcare factors. Utilising the factors identified in this review, future studies should investigate potential strategies to identify at-risk patients and develop new methods to increase persistence and adherence by addressing the modifiable risk factors.

## Supplementary Information

**Additional file 1.**

## Data Availability

Supplementary data sheet available upon request.

## Abbreviations

VEGF: Vascular endothelial growth factor
AMD: Age-related macular degeneration
COVID-19: Coronavirus disease-19
DMO: Diabetic macular oedema
CNV: Choroidal neovascularisation
PRN: Pro re nata
WHO: World Health Organization
PRISMA: Preferred Reporting Items for Systematic Review and Meta-analysis
STATA: Statistical software for data science
MAM: Multidimensional adherence model
RCT: Randomised controlled trial
BCVA: Despite best corrected visual acuity
NIH: National Institute of Health
